# Novel Hydrophobic Ultrafiltration Membranes for Treatment of Oil-Contaminated Wastewater

**DOI:** 10.3390/membranes13040402

**Published:** 2023-04-01

**Authors:** Tatsiana Hliavitskaya, Tatiana Plisko, Alexandr Bildyukevich, Alena Liubimova, Alena Shumskaya, Alexey Mikchalko, Alexandr A. Rogachev, Galina B. Melnikova, Svetlana A. Pratsenko

**Affiliations:** 1 Institute of Physical Organic Chemistry, National Academy of Sciences of Belarus, 220072 Minsk, Belarus; 2 Institute of Chemistry of New Materials, 220141 Minsk, Belarus; 3 F. Skorina Gomel State University, 246019 Gomel, Belarus; 4 Lykov Heat and Mass Transfer Institute, National Academy of Sciences of Belarus, 220072 Minsk, Belarus

**Keywords:** polysulfone, composite membrane, hydrophobization, modification, ultrafiltration, cutting fluid

## Abstract

Cutting fluids are the main source of oily wastewater in the metalworking industry. This study deals with the development of antifouling composite hydrophobic membranes for treatment of oily wastewater. The novelty of this study is that a low energy electron-beam deposition technique was applied for a polysulfone (PSf) membrane with a molecular-weight cut-off of 300 kDa, which is promising for use in the treatment of oil-contaminated wastewater, by using polytetrafluoroethylene (PTFE) as target materials. The effect of the thickness of the PTFE layer (45, 660, and 1350 nm) on the structure, composition, and hydrophilicity of membranes was investigated using scanning electron microscopy, water contact angle (WCA) measurements, atomic force microscopy, and FTIR-spectroscopy. The separation and antifouling performance of the reference and modified membranes were evaluated during ultrafiltration of cutting fluid emulsions. It was found that the increase in the PTFE layer thickness results in the significant increase in WCA (from 56° up to 110–123° for the reference and modified membranes respectively) and decrease in surface roughness. It was found that cutting fluid emulsion flux of modified membranes was similar to the flux of the reference PSf-membrane (7.5–12.4 L·m^−2^·h^−1^ at 6 bar) while cutting fluid rejection (*R_CF_*) of modified membranes increased compared to the reference membrane (*R_CF_* = 58.4–93.3% for modified and *R_CF_* = 13% for the reference PSf membrane). It was established that despite the similar flux of cutting fluid emulsion, modified membranes demonstrate 5–6.5 times higher flux recovery ratio (*FRR*) compared to the reference membrane. The developed hydrophobic membranes were found to be highly efficient in oily wastewater treatment.

## 1. Introduction

Metalworking fluids or cutting fluids are widely used in the engineering industry [1]. Cutting fluids are usually mixed with water in a certain proportion (usually at concentrations of 2–10 wt.%) [2]. The resulting stable emulsions are used to cool, lubricate, and prevent rust in the contact between the processing tool and workpiece being processed. Thus, cutting fluids contribute not only to better processing details, but also to protecting the machining tool from corrosion [3]. Despite a number of advantages of metalworking fluids, it is necessary to mention the disadvantages of their use: cutting fluids are characterized by a rather complex chemical composition, quite dangerous for human health and the environment [4]. Due to their complex chemical composition, cutting fluids are the main source of oily wastewater in the metalworking industry [5]. In particular, it is known, that wastewater contaminated with cutting fluids features high chemical oxygen demand (COD) up to 100,000 mg/L [6,7]. High levels of COD in wastewater can lead to depletion of dissolved oxygen in the water, which in turn will result in negative environmental impacts and regulatory violations [8]. That is why wastewater treatment from cutting fluids is of current interest. Unfortunately, traditional wastewater treatment methods such as flotation, sedimentation, and coagulation do not meet the increased requirements for the quality of wastewater treatment and purity of treated water. Due to this, numerous studies on the use of ultrafiltration (UF) for post-treatment of wastewater from emulsified oil products are currently underway [9,10].

The literature describes the use of various ultrafiltration polymeric membranes of different configuration [11,12,13] from different membrane materials [14,15,16] for the water-oil emulsion separation. In particular, membranes based on polysulfone and polyethersulfone are widely applied for this purpose [14,17,18]. In [17] polysulfone membranes modified with PVP (M_w_ = 24,000–360,000 Da) and PEG (M_w_ = 400–20,000 Da) performed well in the separation of stable oil-in-water emulsions. However, these emulsions were prepared in the laboratory based on surfactant and Crude oil supplied by Guwahati Refinery, Indian Oil Corporation Limited. At the same time, it is known that the composition of emulsions based on cutting fluids is much more complex, which will also affect the ultrafiltration process [19,20]. Although in most cases the composition of a particular cutting fluid is a trade secret, it is widely known that besides petroleum and surfactants, cutting fluids also contain lubricants from natural and synthetic oils, foam, corrosion inhibitors, and other additional compounds [21]. Generally, the scientific literature describes the use of both hydrophilic and hydrophobic membranes for wastewater treatment from emulsified oil products [2,5,9,10,22,23,24]. In particular in [2] commercially available polyvinylidene fluoride (PVDF) hollow fiber UF membranes (molecular weight cut-off (MWCO)—150 kDa) were successfully used for filtration of concentrated emulsion based on commercial cutting fluid, ULTRACUT^®^ EVO 260 (ROCOL, UK). In [9,25,26] it is reported that PVDF ultrafiltration membranes are quite often used for separation emulsions based on cutting fluids, moreover, PVDF-based membranes are one of the most used materials for this purpose. For example, [25] reports the preparation of a TiO_2_/poly(vinylidene fluoride–trifluoro ethylene) nanocomposite membrane by solvent casting and its successful photocatalytic application in the remediation of oily wastewater. In [26] the authors report on the development, characterization, and application of a PVDF—hexafluoropropylene porous membrane which was proved to be suitable for wastewater treatment in order to remove of organic matter. At the same time, PVDF-membranes may also have certain limitations for the separation of emulsions stabilized by surfactants, especially if the pore size of the membrane is large, as reported in [21]. In [22] for oil-water emulsion separation hydrophilic regenerated cellulose ultrafiltration membranes modified by grafting poly(N-isopropylacrylamide)-block-poly(oligoethylene glycol methacrylate) nanolayers were used. Cellulose membranes for oil-water emulsion separation have also been used in [23]. At the same time, in [24] it is noted that the use of hydrophilic ultrafiltration membranes for oil-water emulsion separation is undesirable, since the flux of such membranes can easily be reduced due to clogging of the membrane pore and fouling of the surface by the oil. In [9,10] it is postulated that the hydrophobization of the selective layer of polymer membranes contributes to the effective purification of wastewater from cutting fluids due to the specific interaction between the membrane and emulsion components. At the same time, there are very few works devoted to the hydrophobization of the selective layer of polymeric membranes in order to separate emulsions of cutting fluids. In the articles devoted to this topic, silica [27,28,29,30] nanoparticles are incorporated in the matrix of porous polymer membrane for preparation of hydrophobic membranes. In [29] 0.5–3.0 wt% modified hydrophobic nano-SiO_2_, with an average diameter of 20 nm was added in PVDF/N,N-dimethylformamide (DMF) solution under the ultrasonic stirring, which led to an increase in the water contact angle from 138.5° up to 150°. In [30] microfiltration PVDF membranes were modified by their immersing in a PVDF/DMF solution, which additionally contained specially fabricated SiO_2_ nanoparticles modified with hexamethyldisilazane. Water contact angle of such membranes reached 158°. A rather difficult task during preparation of such membranes is the uniform dispersion of nanoparticles in the casting solution due to their tendency to aggregation [31,32]. Often, this problem cannot be solved without additional surface modification of nanoparticles [31,33]. Moreover, this significantly complicates the process of membrane modification. In this regard, the development of alternative methods for membrane modification in order to improve their separation performance during ultrafiltration of cutting fluids is very relevant. One more method for obtaining hydrophobic membranes for separation of cutting fluid emulsions is the manufacture of polytetrafluoroethylen (PTFE) or PVDF membranes via the electrospinning technique [34,35,36]. In [37] it is indicated that the industrial scale-up of the process of membrane preparation by electrospinning technique is restricted by limited reproducibility and low production capacity. The literature also describes an alternative method of hydrophobization of the surface of the selective layer of membranes—electron-beam sputtering of polytetrafluoroethylene in a vacuum [38,39,40,41]. In particular, in [38] it is considered that electron-beam sputtering of PTFE onto the surface of the poly(ethylene terephthalate) track-etched membrane leads to their effective hydrophobization, the contact angle increases from 65 up to 110–120^o^. Membrane modification by electron beam deposition is mainly carried out to hydrophobize the selective layer for the application of membranes in membrane distillation processes for desalination of seawater [40,41]. It was revealed that there is practically no information in the literature on the use of the electron-beam sputtering technique in relation to the hydrophobization of membranes used for wastewater purification from cutting fluids.

In [42,43] the results of application of low-energy (0–100 eV) electron beams for the deposition induced by a focused electron beam of thin fibers or low-molecular-weight compounds are shown. However, to intensify the process, electric beams with energies above 500 eV are used [44]. In the series of our previous studies devoted to the formation of thin-layer coatings consisting of polymers, polymer-metal compositions, polymer-organic compounds (drugs), we investigated the possibility of using high molecular weight compounds (without destroying their structure) for electron beam deposition with a flow rate of 800–1600 eV [41,45,46]. So, the novelty of this study is the application of a low energy electron beam for deposition of a high molecular compound—PTFE without destroying its polymer structure. On the other hand, the novelty is in the application of this method for development of composite membranes with a hydrophobic surface for a specific task—treatment of oily contaminated wastewater. It was expected that membrane modification will improve the hydrophobic properties of the initial membranes which will yield an increase in the efficiency of purification of wastewater from cutting fluids. Thereby, the aim of this work is to study the effect of physicochemical modification of polysulfone (PSf) membranes by PTFE via low energy electron-beam deposition technique on the pore structure and physico-chemical properties of the selective layer as well as the separation and antifouling performance of membranes in wastewater treatment from metalworking fluids.

## 2. Experimental

### 2.1. Materials

Commercially available polysulfone flat-sheet ultrafiltration membranes with molecular weight cut-off (MWCO) of 300 kDa (PSf-300) were used as the initial membranes for modification in this study. The PSf-300 membrane was developed and produced by the Institute of Physical Organic Chemistry, National Academy of Sciences of Belarus (Minsk, Republic of Belarus). A 2 wt.% water emulsion of commercial cutting fluid SOZH VITTOL–297, (Vittol 297, density less than 920 kg/m^3^ at 20 °C, SERVOVIT, Minsk, Belarus) was used for ultrafiltration experiments to evaluate membrane performance and antifouling stability. The cutting fluid, SOZH VITTOL-297, was prepared on the basis of petroleum oils with the addition of surfactants, corrosion inhibitors, and bactericides. This cutting fluid is an analogue of such cutting fluids as Rhenus FU 51, and Mobilcut 230. Concentration of oil content 2 wt.% was selected on the basis of cutting fluid SOZH VITTOL-297 instructions for use. The 2 wt. % SOZH VITTOL-297 water emulsion is used for grinding steel and cast iron, as well as for turning, drilling, milling, steel and non-ferrous metals.

### 2.2. Membrane Modification

**Figure 1:**
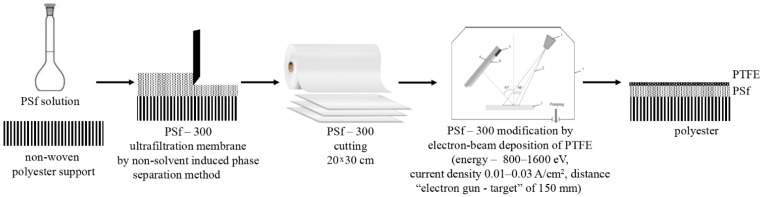
The principal schema of creation and modification of ultrafiltration polysulfone membrane.

**Figure 2:**
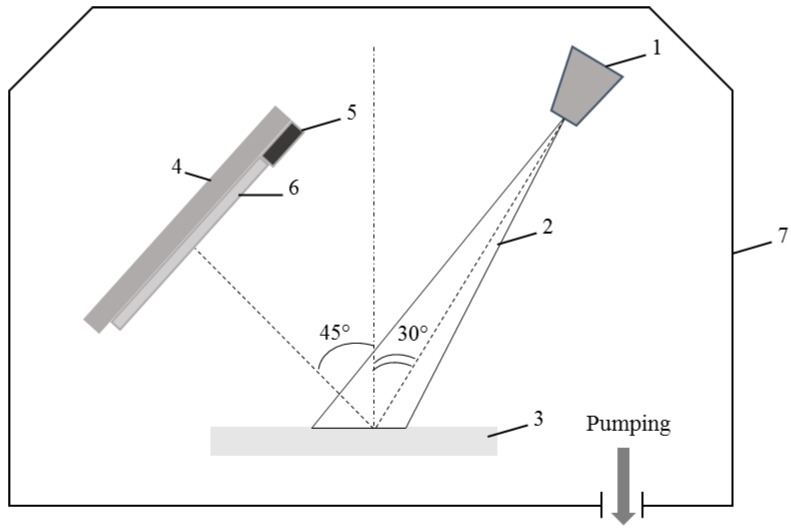
Scheme of low energy electron-beam deposition device: 1—electron gun, 2—electron beam, 3—PTFE target, 4—substrate holder, 5—quartz crystal microbalance device, 6—membrane, 7—vacuum chamber.

Modification of commercial ultrafiltration polysulfone membrane PSf-300 (M_o_) was carried out. The principal schema of creation and modification of ultrafiltration polysulfone membrane are on the Figure 1 and Figure 2.

The scheme of the device for electron-beam deposition of a coating based on PTFE in a vacuum is shown in Figure 2. In the electron gun, free electrons are emitted from the cathode surface and formed under the action of accelerating and focusing electrostatic and magnetic fields into a beam, which is led into the working chamber through the outlet (1). The electron beam (2) modeled with the help of magnetic focusing lenses and deflecting systems in high vacuum (7) passes unhindered to the target made of the material to be sputtered, in our case a PTFE plate (3). Due to the bombardment of the surface with an electron beam, the material is heated to a temperature at which it evaporates at the required rate. A substrate fixed on a holder (4), in our case, an ultrafiltration membrane PSf-300 (6), on which the evaporated substance is condensed, is placed in the resulting flow. The evaporator is supplemented with measurement and control devices (5), which are especially important for controlling the electron beam during the deposition process. During deposition on the membrane PSf-300, a layer of PTFE is formed, first in the form of islands, which grow to form a constant uniform layer. A low energy electron-beam deposition was performed at the following process parameters: an energy of 800–1600 eV, a current density of 0.01–0.03 A/cm^2^, pressure of residual gases in a vacuum chamber of ≈ 4 × 10^−3^ Pa, the average distance “electron gun-target” of 150 mm. The thickness of the layer was controlled with a quartz crystal microbalance device. The thickness of the PTFE layer reached 45 (M_1_), 660 (M_2_), and 1350 (M_3_) nm. Membrane abbreviations according to the thickness of the modifying PTFE layer are presented in Table 1. PTFE was chosen due to its well-known high hydrophobic properties [38].

### 2.3. FTIR Analysis

The FTIR (Nicolet Is50 spectrometer, Thermo Fisher Scientific, Waltham, MA, USA) spectra of the initial and modified membranes were used to study the chemical composition of the selective layer of membranes. Prior to measurements, the membranes were rinsed several times with distilled water and then dried for 48 h.

### 2.4. Membrane Structure Studies

The structures of the initial and modified-by-PTFE membranes were investigated using a scanning electron microscope (SEM) Phenom Pro (PhenomWorld scanning electron microscopes, Thermo Fisher Scientific, Waltham, MA, USA). The samples were previously broken up in liquid nitrogen, and thereafter a 10 Å thick layer of gold was deposited on the membrane samples by a vacuum sputter coater DSR (Vaccoat, London, UK).

The topography of the selective layer surface was studied by using atomic force microscopy (AFM NT-206, Microtestmashines, Gomel, Belarus). Standard silicon cantilevers (NSC35, MikroMasch, Wetzlar, Germany) with a rigidity 3.5 N/m (according to the manufacturer’s specification) were used for this study.

### 2.5. Water Contact Angle Measurements

Water contact angles (WCA) were determined from the “membrane-0.02 M NaCl-air” system as described in [47]. Membranes were previously fixed in a water-salt solution in such a way that the selective layer was oriented downwards. An air bubble with a constant volume 0.01 cm^3^ was placed in the membrane selective layer by using a special dispenser. A goniometer instrument LK-1 (Open Science, Moscow, Russia) was used to record the water contact angles.

### 2.6. Flux, Cutting Fluid Rejection, and Antifouling Performance of the Membranes

The pure water fluxes (PWF, *J*_0_, L·m^−2^·h^−1^) for all studied membranes were determined in a dead-end ultrafiltration cell with a surface area of 23 cm^2^. The ultrafiltration cell was equipped with a magnetic stirrer (rotation speed 250 rpm). The ultrafiltration tests were conducted at a transmembrane pressure (TMP) of 1 bar. To evaluate PWF all membranes were previously cleaned with an alkaline cleaning solution (1.0 wt.% Ultrasil 10, Ecolab, Stockholm, Sweden) for 1 h at 50 °C. Membrane fluxes were calculated as follows:

(1) $$J_{X}=\frac{V}{\left(S\times t\right)}$$

where *V* (L) is the volume of permeate; *S* (m^2^) is the working area of the membrane surface; *t* (h) is the filtration time.

Fluxes for the 2.0 wt.% cutting fluid solution (*J_CF_*) were measured after 40 min of filtration of this solution at a transmembrane pressure (TMP) of 1–7 bar. To determine membrane cutting fluid rejections (*R_CF_*) UV spectrophotometer (Metertech UV–VIS SP 8001, Metertech, Taipei, Taiwan) at 500 nm wavelength was used, as described in [48,49]. *R_CF_* were calculated as follows:

(2) $$R_{CF}=\left(1-\frac{C_{p}}{C_{f}}\right)\times 100\%$$

where *C_p_* represents cutting fluid content in the permeate, and *C_f_* represents cutting fluid content in the feed solution.

During ultrafiltration, experiment flux recovery ratios (*FRR_CF_*, %), reversible flux decline ratio (*DR_r_*), and irreversible flux decline ratio (*DR_ir_*) were estimated according to Equations (3)–(5):

(3) $${FRR}_{CF}=\frac{J_{0CF}}{J_{0}}\times 100\%$$

(4) $${DR}_{r}=(\frac{J_{0CF-}J_{CF}}{J_{0}})\times 100\%$$

(5) $${DR}_{ir}=(\frac{J_{0-}J_{0CF}}{J_{0}})\times 100\%$$

where

*J*_0_—pure water flux at TMP 1 bar, L·m^−2^·h^−1^

*J_CF_*—flux of 2.0 wt.% cutting fluid emulsion at 5 bar, L·m^−2^·h^−1^

*J*_0_*_CF_*—pure water flux at TMP 1 bar after ultrafiltration of cutting fluid emulsion, L·m^2^·h^−1^

The experiment was set up as follows: after rinsing membranes with distilled water for 40 min PWF of such membranes (*J*_0_) were measured at room temperature 20 °C and TMP of 1.0 bar, after which a 2.0 wt.% cutting fluid aqueous emulsion was filtered through the membrane for 40 min under TMP of 5.0 bar. Thereafter, the flux of cutting fluid emulsion (*J_CF_*) was measured. Measurements were taken at 40 min, because by this time the performance reaches a steady state mode of filtration of cutting fluid solution which is proved by the fact that flux is kept constant and does not change over the time. After this, the feed solution was replaced with distilled water and PWF of such membranes (*J*_0*CF*_) were measured again, at room temperature 20 °C and TMP of 1 bar.

## 3. Results and Discussion

### 3.1. Study of the Effect of PTFE-Modification on Composition of Membrane Selective Layer

**Figure 3:**
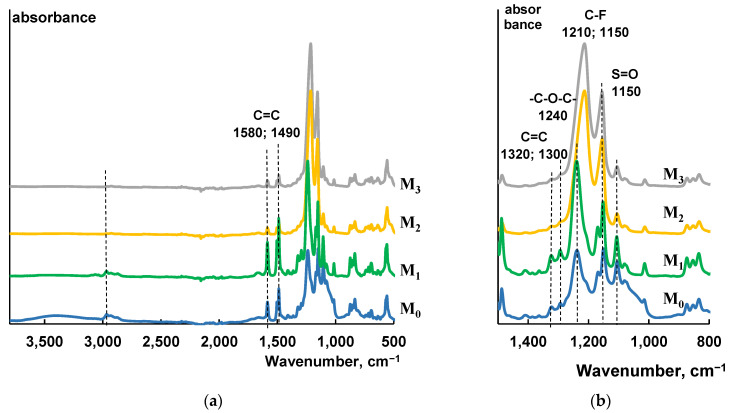
FTIR spectra (**a**) and fragment of FTIR spectra (**b**) of initial PSf-membrane and membranes modified with PTFE.

The FTIR spectra of the selective layer surface of the initial (M_0_) and modified PSf-membranes (M_1_–M_3_) are presented in Figure 3. For the initial polysulfone membrane the vibrations of S=O bonds were observed at 1320, 1300, and 1150 cm^−1^ [50]. The IR peaks at 1580 and 1490 cm^−1^ are related to C=C bonds of the aromatic ring of the PSf. The peak observed at 2960 cm^−1^ belongs to the vibration band of =C–H in the aromatic ring of polysulfone. The absorption peak at 1240 cm^−1^ corresponds to the stretching vibrations of –C–O–C– in the ether group [50]. FTIR spectra of PSf membranes modified with a PTFE layer with the thickness of 660 and 1350 nm are characterized by two typical transmittance bands of C-F bond (asymmetric stretching vibration at 1210 cm^−1^ and symmetrical stretching vibration at 1150 cm^−1^) [51].

These results of FTIR spectroscopy studies indicate successful modification of PSf membranes by PTFE. It was confirmed that selective layers of modified membranes mainly consist of PTFE, and the peaks characteristic of polysulfone are not detected. In the case of M1 membranes modified with PTFE (thickness of modified layer 45 nm), a significant increase in the intensity of peaks in the region of 1150 and 1240 cm^−1^ are detected, which is also a confirmation of the successful modification of membranes with PTFE. However, some peaks which are assigned to PSf (1580 and 1490 cm^−1^) are also present which proves that there is not a complete overlapping of the membrane surface by the PTFE layer.

### 3.2. Effect of PTFE-Modification on the Structure of Membranes

**Figure 4:**
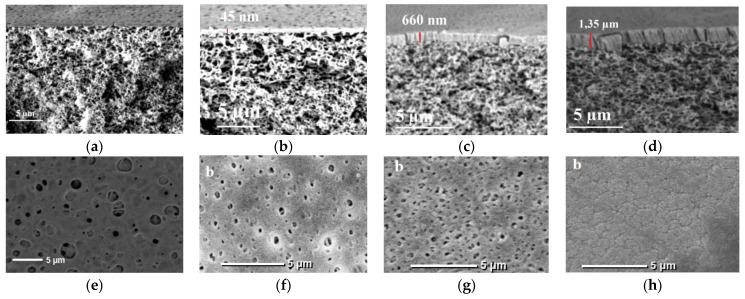
SEM images of the cross-section structure (**a**–**d**) and selective layer surface (**e**–**h**) of initial PSf membrane M_0_ (**a**,**e**), and modified M_1_ (**b**,**f**), M_2_ (**c**,**g**), and M_3_ (**d**,**h**) membranes.

Successful PSf-membranes modification with PTFE by a low energy electron-beam deposition technique was confirmed by the results of scanning electron microscopy studies, see Figure 4. The thickness of the PTFE-layer reached 45 nm for M_1_, 660 nm for M_2_, and 1350 nm for M_3_, whichwas proved using SEM.

It was found that an increase in the thickness of the modifying PTFE-layer leads to the gradual overlapping of pores on the surface of the selective layer. Significantly reduced longitudinal and transverse pore size was revealed for modified membranes. The thicker the selective layer, the smaller the pore size on the membrane surface. Thus, for the initial membrane M_0_, the pore size on the surface of the selective layer is quite large. Modification of membranes with a PTFE-layer 45 nm thick (membrane M_1_) leads to a significant reduction in the pore size on the surface of the selective layer down to 300–500 nm. A further increase in the thickness of the modifying PTFE-layer up to 660 nm (membrane M_2_) leads to a further decrease in the pore size down to 150–300 nm. However, it was found that a significant thickness of the PTFE-coating up to 1350 nm (membrane M_3_) does not yield the complete overlapping of pores, leaving microcracks and breaks on the membrane surface, Figure 4h.

**Figure 5:**
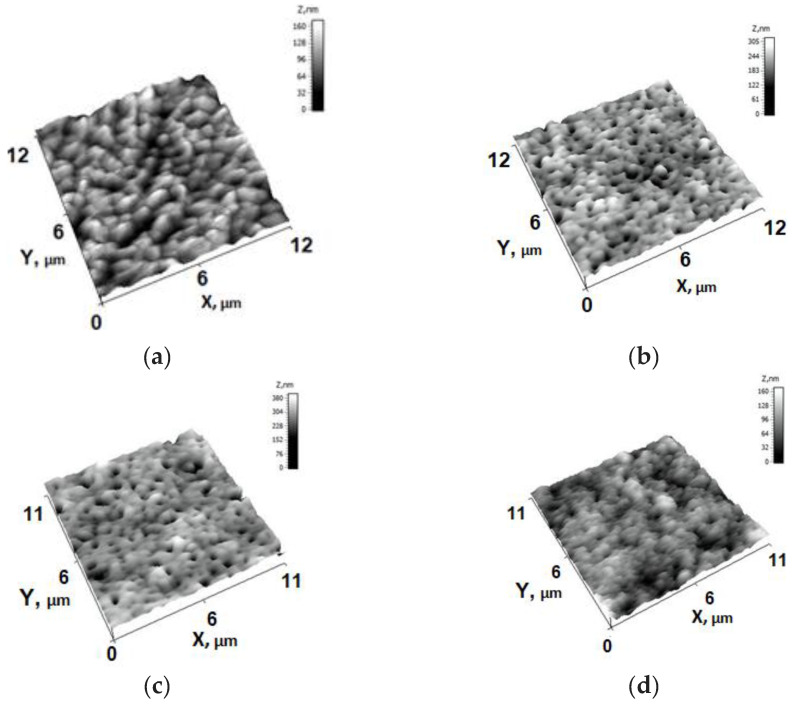
AFM micrographs of skin layer of initial PSf-membrane M_0_ (**a**) and membranes modified with PTFE-layer thickness 45 nm (M_1_) (**b**), 660 nm (M_2_) (**c**), and 1350 nm (M_3_) (**d**).

Figure 5 shows the AFM-images of the initial PSf 300 (M_0_) and PTFE-modified membranes (M_1_–M_3_). The bright areas indicate the highest points of the membrane surface and the dark regions indicate valleys of the membrane. It was revealed that the deposition of the PTFE layer significantly changes the topography of the surface of the membrane selective layer, Figure 5. The surface of the PSf 300 membrane (M_0_) features numerous structural elements with sharp ridges which result in relatively high surface roughness parameters (average surface roughness (R_a_) and root-mean-squared surface roughness (R_q_) (Table 2). The elongated valleys with the length of up to 1000 nm between these edges are not deep. It was found that the deposition of the PTFE layer leads to turning sharp ridges into globular structural elements with deep round valleys between them (the valleys can contain pores at the bottom). The increase in PTFE layer thickness results in the increase in the size of globular structural elements on the membrane surface and a decrease in the number of valleys. For modified membranes (M_1_–M_3_), structural elements are evenly distributed over the membrane surface and are characterized by a significantly smaller size compared to the initial one (the diameter of valleys for modified membranes does not exceed 500 nm, while in the case of the initial membrane the diameter of such valleys reaches 1000 nm). It was established that the modification of PSf-membranes with PTFE leads to a slight decrease in membrane surface roughness. The electron-beam deposition of PTFE on the PSf-membrane surface leads to a slight decrease in average roughness (R_a_) and root-mean-squared surface roughness (R_q_) from 39.3 down to 30.4–15.7 nm and from 50,8 down to 40.1–19.2 nm respectively, see Table 2.

It was shown that water contact angle significantly increased from 56° for M_0_ up to 110–120^o^ for M_1_–M_3_. Such high values of WCA also indicate the successful modification of PSf membranes with hydrophobic PTFE.

### 3.3. Effect of Modification by Low-Energy Electron Beam Deposition of PTFE on Membrane Permeability

**Figure 6:**
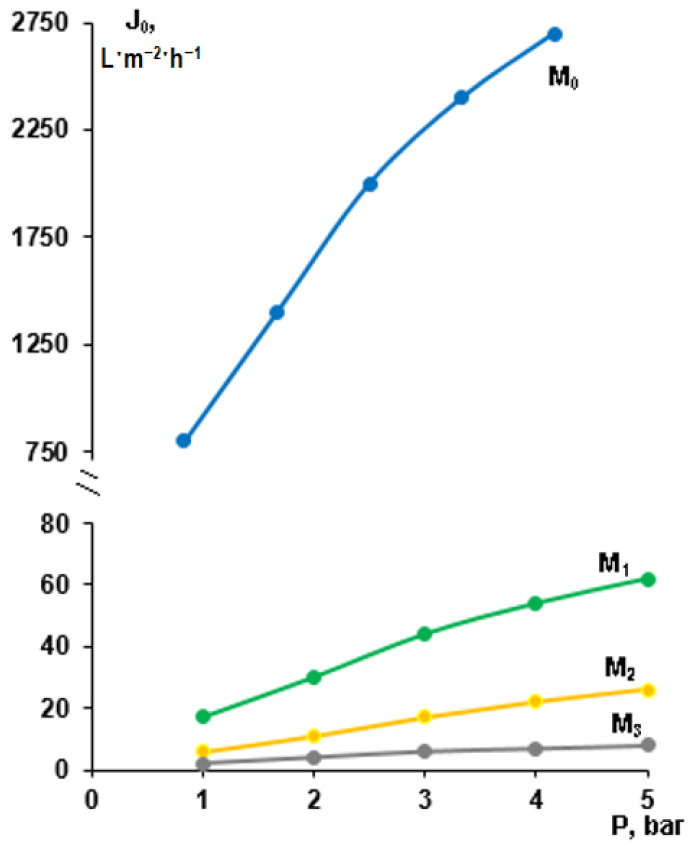
Pure water fluxes for initial PSf membrane (M_0_) and membranes modified with PTFE-layer thickness 45 nm (M_1_), 660 nm (M_2_), and 1350 nm (M_3_).

From Figure 6 it can be seen that the modification of PSf 300 membrane by low energy electron-beam deposition of PTFE significantly affects the membrane PWFs. The PWFs of initial PSf-membranes (M_0_) are in range of 800–2700 L·m^−2^·h^−1^ depending on transmembrane pressure. It was found that for the initial PSf membrane (M_0_) when transmembrane pressure increases from 1 bar to 3 bar the PWF increases linearly from 800 to 2000 L·m^−2^·h^−1^. However, when transmembrane pressure exceeds 3 bar the dependence of PWF on pressure deviates from the linear trend. This tendency can be also observed for modified M_1_, M_2_, and M_3_ membranes. Such deviation from the linear dependence “Pure water flux–pressure” can be explained by the compaction of the porous structure of the membranes [52,53].^.^ It was found that electron-beam deposition of PTFE leads to a significant decrease in membrane PWF. For example, PWF of initial PSf membrane (M_0_) was 2700 L·m^−2^·h^−1^ (at 5 bar), for membrane M_1_ modified with PTFE layer thickness 45 nm PWF was 67 L·m^−2^·h^−1^ (at 5 bar), for membrane M_2_ PWF was 26 L·m^−2^·h^−1^ (at 5 bar), and for membrane M_3_ PWF it was only 8 L·m^−2^·h^−1^ (at 5 bar). It was found that an increase in the thickness of the PTFE selective layer yields a decrease in the pore size of the selective layer, Figure 4, which results in a dramatic drop of PWF. Moreover, the additional layer of PTFE not only overlaps the pores of the selective layer but also significantly increases the selective layer thickness and creates an additional barrier to mass transfer through the membrane. Such a significant decrease in membrane PWF can be explained not only by a change in the structure of the selective layer of modified membranes, as a result of additional sputtering of the PTFE layer, but also by a significant increase in the values of the water contact angles of modified membranes from 56 up to 110–120° (Table 2). It is widely known that membrane hydrophobization prevents water molecules from entering membrane pores and decreases membrane water permeability.

### 3.4. Cutting Fluid Emulsion Ultrafiltration Experiment—Fluxes and Retentions

**Figure 7:**
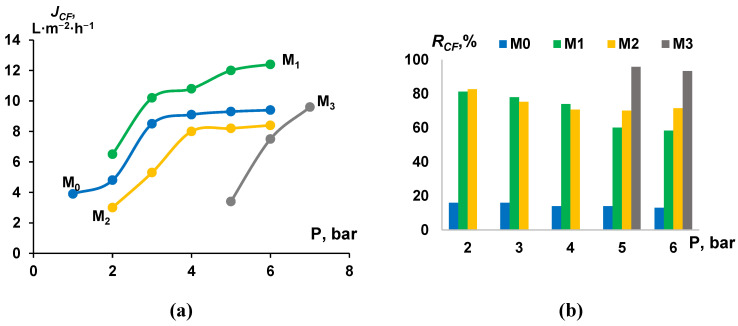
Dependence of 2 wt.% cutting fluid emulsion flux (**a**) and cutting fluid rejection (**b**) on transmembrane pressure during ultrafiltration.

The ultrafiltration of cutting fluid emulsions was carried out using initial and modified-by-PTFE membranes (see Figure 7a). Despite the significant difference in PWF for the initial and modified membranes, all membranes during ultrafiltration of cutting fluids emulsions showed comparable values of fluxes (2–12.4 L·m^−2^·h^−1^ depending on the transmembrane pressure). This improved performance of the modified membranes (M_1_–M_3_) despite much lower PWF compared to the reference membranes (M_0_) is due to their increased hydrophobicity. According to [9,10] hydrophobization of the selective layer of polymer membranes contributes to the effective purification of wastewater from cutting fluids. The permeability of membranes increased each time the pressure was increased: from 3.9 L·m^−2^·h^−1^ (at 1 bar) to 9.4 L·m^−2^·h^−1^ (at 6 bar) for initial membrane (M_0_) and from 6.5 L·m^−2^·h^−1^ (at 2 bar) to 12.4 L·m^−2^·h^−1^ (at 6 bar) for the membrane M_1_ modified with PTFE-layer thickness 45 nm (Figure 7a). Membrane M_3_ modified with PTFE-layer of 1350 nm thickness was impermeable up to a pressure of 5 bar, after which it also showed comparable performance values 7.5 L·m^−2^·h^−1^ (at 6 bar). Despite the similar fluxes during ultrafiltration of cutting fluid emulsion, membranes modified with PTFE demonstrate much higher cutting fluid rejection (*R_CF_*) (Figure 7b). For initial membrane M_0_ the cutting fluid rejection was in the range of 13–16% depending on TMP, while in the case of the modified membranes M_1_–M_3_ the cutting fluid rejection increased significantly. For example, for membrane M_0_ cutting fluid rejection was in the range of up to 58.4–81.2%, depending on TMP. The highest values of cutting fluid rejection (83.3–95.8%) were registered for membranes M_3_ modified with PTFE-layer with the thickness of 1350 nm. Thus, there is a tendency to increase the values of the cutting fluid rejection of membranes with an increase in the thickness of the deposited PTFE-layer. At the same time, it was found that with an increase in TMP, a decrease in cutting fluid rejection occurs. The most significant decrease in cutting fluid rejection (from 81.2% at 2 bar down to 58.4% at 6 bar) was recorded for membranes M_1_ modified with PTFE-layer with the thickness of 45 nm.

**Figure 8:**
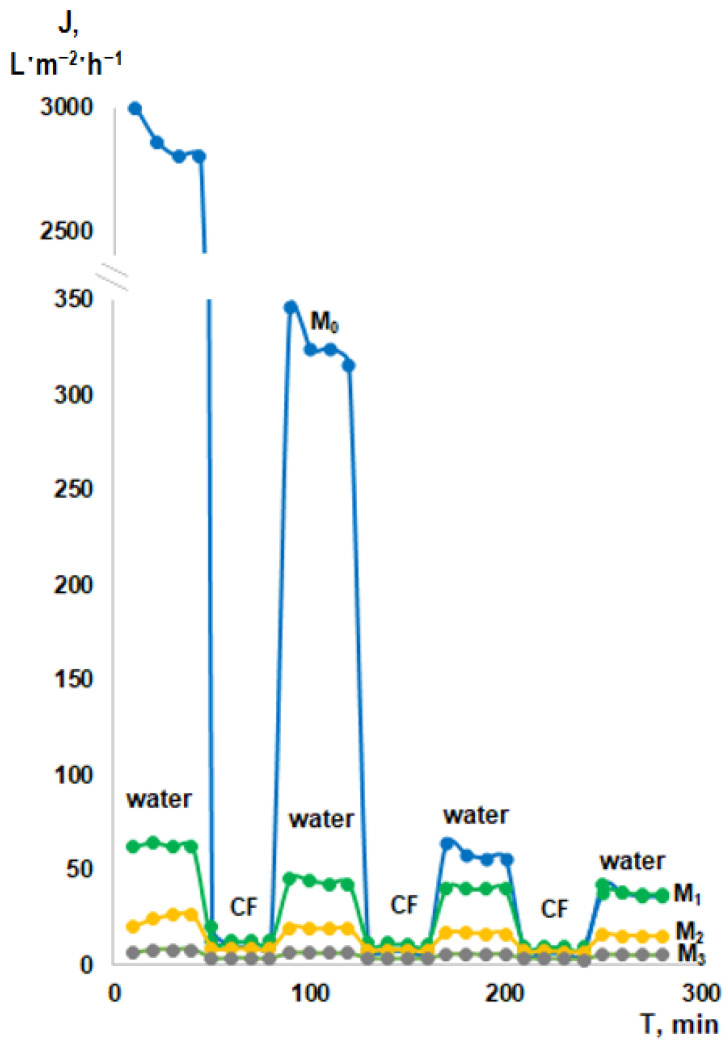
Dependence of permeation flux on the time during ultrafiltration water and 2 wt.% cutting fluid emulsion.

**Figure 9:**
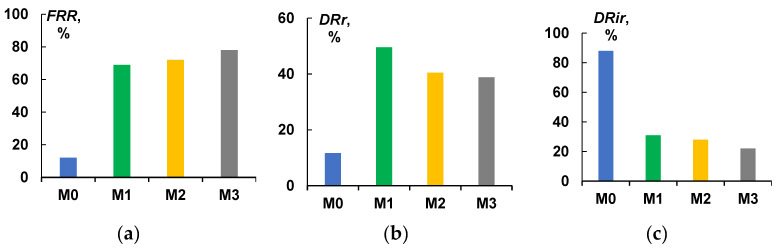
Fouling parameters of PSf-membranes: (**a**) flux recovery ratio (*FRR*); (**b**) reversible flux decline ratio (*DR_r_*), and (**c**) irreversible flux decline ratio (*DR_ir_*).

The increased rejection coefficients of cutting fluids suggested that PTFE-modified membranes were promising for wastewater treatment from emulsified oil products. Furthermore, during the long-term ultrafiltration experiment of cutting fluid solution, it was found that PWF of initial unmodified membrane M_0_ decreased extremely after each cycle of cutting fluid emulsion ultrafiltration, while for the modified-with-PTFE membranes M_1_–M_3_ there is no such significant drop in PFW, see Figure 8. The long-term experiment was set up as follows: Every 40 min the water was replaced with a cutting fluid solution. Flux of membranes was recorded every 10 min at room temperature 20 °C and TMP of 5.0 bar. For example, PWF of the initial membrane M_0_ before ultrafiltration of the cutting fluid emulsion was in the range of 3000–2700 L·m^−2^·h^−1^, while after the first ultrafiltration cycle of cutting fluid emulsion PWF decreased extremely down to 350–330 L·m^−2^·h^−1^, after the second ultrafiltration cycle of cutting fluid emulsion PWF was in the range 55–65 L·m^−2^·h^−1^, and after the third cycle of ultrafiltration cutting fluid emulsion PWF was only 30–35 L·m^−2^·h^−1^, which was equal to PWF of modified-with-PTFE membrane. At the same time, the PWF of the modified membranes M_1_–M_3_ does not change significantly after each cycle of cutting fluid emulsion ultrafiltration, see Figure 8. It should be noted that the initial PSf-membrane (M_0_) is characterized by rather high values of average roughness (R_a_) and root-mean-squared surface roughness (R_q_), as well as rather low values of water contact angle (56 ± 2°). Apparently, the combination of these factors affects the rather high fouling of the membrane with cutting fluids, which is manifested by a significant drop in the membrane flux in the case of replacing water with cutting fluid solution. At the same time, modified membranes (M_1_–M_3_), on the contrary, are characterized by a hydrophobic surface (WCA = 110–120°) and much lower average roughness (R_a_) and root-mean-squared surface roughness (R_q_) values. Therefore, such a dramatic decrease in flux is not recorded, as in the case of the initial M_0_ membrane. This statement is also confirmed by the values of flux recovery ratios, see Figure 9a. The apparently hydrophobic PTFE-layer characterized by low roughness, as well as the presence of surfactants in the cutting fluid emulsion, contributes to a significant enhanced removal of pore fouling material in case of modified membranes (M_1_–M_3_) [9,10].

The flux recovery ratios for the initial M_0_ membrane during the ultrafiltration of cutting fluid emulsion tests was relatively low (*FRR* = 11.7%), as for PTFE-modified membranes M_1_–M_3_ significant increase in *FRR* was recorded (up to 69–78%). Modifications of the membrane surface with PTFE leads to the enhancement of antifouling performance of membranes. Moreover, an increase of PTFE layer thickness results in the increase in fouling recovery ratio, see Figure 9a.

The modified-with-PTFE membranes M_1_–M_3_ were characterized by increased resistance to fouling in comparison with the initial PSf membrane M_0_. The modification with PTFE leads to an increase in the reversible flux decline ratio of the membranes from 11.7 up to 38.8–49.6% (Figure 9b) and a decrease in the irreversible flux decline ratio (*DR_ir_*) from 88.0 down to 22.1–31.0% (Figure 9c), which promotes more efficient removal of the fouling from the selective surface of the membranes. It is known that membrane antifouling performance depends on membrane pore size, surface roughness, hydrophilicity, and charge of the selective layer. Modification with PTFE leads to the decrease in membrane pore size and surface roughness parameters which both yield the increase in resistance to membrane fouling. However, a significant increase in membrane water contact angle does not result in the decrease to antifouling performance which may be attributed to peculiarities of the material of the membrane selective layer of the modified membranes—PTFE which is inert and features low wettability by water and oils [54,55].

## 4. Conclusions

A novel method of flat sheet polymeric membranes modification in order to hydrophobize the selective layer was proposed. Modification was carried out using a low energy electron-beam deposition technique by using PTFE as a target material, to improve the hydrophobic properties of the initial membranes. The successful modification of the membrane’s selective layer was confirmed by SEM and AFM-results as well as FTIR spectroscopy. Deposition of a PTFE-layer on the membrane surface leads to significant increase in water contact angle of the membranes from 56° up to 110–123°. In turn, an increase in hydrophobicity, decrease in pore size, and formation of an additional barrier layer led to a decrease in PWF of the modified membranes from 800 down to 2–17 L·m^−2^·h^−1^. Comparisons of the initial membrane with the modified membranes were carried out using 2,0 wt.% cutting fluid aqueous emulsion as feed solution during ultrafiltration in order to purify water from emulsified oil products. It was established that the flux for initial and modified membranes was in the range of 2–12.9 L·m^−2^·h^−1^, while rejection of cutting fluid was different: 14–16% for initial PSf-membrane and 60.1–95.8% for modified-with-PTFE membranes. Modified membranes exhibit higher flux and 5–6 times higher membrane flux recovery ratio. Thus, the developed PSf-PTFE membrane was found to be highly efficient for oil-contaminated wastewater purification.

## Figures and Tables

**Table 1:** Membrane abbreviations depending on the thickness of the modifying PTFE layer.

Membrane Abbreviation	Modifying PTFE Layer Thickness (nm)
M_0_	0
M_1_	45
M_2_	660
M_3_	1350

**Table 2:** Surface roughness parameters and water contact angles of membranes.

Membrane Abbreviation	PTFE Layer Thickness, (nm)	R_a_ (nm)	R_q_ (nm)	WCA (°)
M_0_	0	39.3	50.8	56 ± 2
M_1_	45	30.4	40.1	110 ± 2
M_2_	660	28.8	36.0	120 ± 2
M_3_	1350	15.7	19.2	120 ± 2

## Data Availability

Data available on request. The data presented in this study are available on request from the corresponding authors.

## References

[1] MacAdam J., Ozgencil H., Autin O., Pidou M., Temple C., Parsons S., Jefferson B. (2012). Incorporating biodegradation and advanced oxidation processes in the treatment of spent metalworking fluids. Environ. Technol..

[2] Palanisamy T., Tabatabai S.A.A., Zhang T., Leiknes T. (2021). Role of surfactants in cleaning of PVDF ultrafiltration membranes fouled by emulsified cutting oil. J. Water Process Eng..

[3] Sales W.F., Diniz A.E., Machado Á.R. (2001). Application of cutting fluids in machining processes. J. Braz. Soc. Mech. Sci..

[4] Debnath S., Reddy M.M., Yi Q.S. (2014). Environmental friendly cutting fluids and cooling techniques in machining: A review. J. Clean. Prod..

[5] Amin M.M., Mofrad M.M.G., Pourzamani H., Sebaradar S.M., Ebrahim K. (2017). Treatment of industrial wastewater contaminated with recalcitrant metal working fluids by the photo-Fenton process as post-treatment for DAF. J. Ind. Eng. Chem..

[6] Vahid A., Mojtaba F., Abbas S., Reza K. (2013). Evaluation of the Metalwork Cutting Fluid Treatment performance Using Fenton Oxidation Process in Comparison with Coagulation-Flocculation. Casp. J. Appl. Sci. Res..

[7] Seo D.C., Lee H.J., Hwang H.N., Park M.R., Kwak N.W., Cho I.J., Heo J.S. (2007). Treatment of non-biodegradable cutting oil wastewater by ultrasonication-Fenton oxidation process. Water Sci. Technol..

[8] Liu X., Wang D., Li A. (2021). Biodiesel production of Rhodosporidium toruloides using different carbon sources of sugar-containing wastewater: Experimental analysis and model verification. J. Clean. Prod..

[9] Rasouli S., Rezaei N., Hamedi H., Zendehboudi S., Duan X. (2021). Superhydrophobic and superoleophilic membranes for oil-water separation application: A comprehensive review. Mater. Des..

[10] Lopez-Torres D., Elosua C., Hernaez M., Goicoechea J., Arregui F.J. (2015). From superhydrophilic to superhydrophobic surfaces by means of polymeric layer-by-layer films. Appl. Surf. Sci..

[11] Choong L.T.S., Lin Y.M., Rutledge G.C. (2015). Separation of oil-in-water emulsions using electrospun fiber membranes and modeling of the fouling mechanism. J. Membr. Sci..

[12] Al-Malack M.H. (2013). Treatment of petroleum refinery wastewater using crossflow and immersed membrane processes. Desalin. Water Treat..

[13] Li Q., Yan Z.Q., Wang X.L. (2016). A poly (sulfobetaine) hollow fiber ultrafiltration membrane for the treatment of oily wastewater. Desalin. Water Treat..

[14] Chen W., Peng J., Su Y., Zheng L., Wang L., Jiang Z. (2009). Separation of oil/water emulsion using Pluronic F127 modified polyethersulfone ultrafiltration membranes. Sep. Purif. Technol..

[15] Ochoa N.A., Masuelli M., Marchese J. (2003). Effect of hydrophilicity on fouling of an emulsified oil wastewater with PVDF/PMMA membranes. J. Membr. Sci..

[16] Wang X., Fang D., Yoon K., Hsiao B.S., Chu B. (2006). High performance ultrafiltration composite membranes based on poly (vinyl alcohol) hydrogel coating on crosslinked nanofibrous poly (vinyl alcohol) scaffold. J. Membr. Sci..

[17] Chakrabarty B., Ghoshal A.K., Purkait M.K. (2008). Ultrafiltration of stable oil-in-water emulsion by polysulfone membrane. J. Membr. Sci..

[18] Chakrabarty B., Ghoshal A.K., Purkait M.K. (2010). Cross-flow ultrafiltration of stable oil-in-water emulsion using polysulfone membranes. Chem. Eng. J..

[19] Jagadevan S., Jayamurthy M., Dobson P., Thompson I.P. (2012). A novel hybrid nano zerovalent iron initiated oxidation–Biological degradation approach for remediation of recalcitrant waste metalworking fluids. Water Res..

[20] Muszyński A., Załęska–Radziwiłł M., Łebkowska M., Nowak D. (2007). Biological and electrochemical treatment of used metalworking fluids: A toxicity-reduction evaluation. Arch. Environ. Contam. Toxicol..

[21] Zhang W., Shi Z., Zhang F., Liu X., Jin J., Jiang L. (2013). Superhydrophobic and superoleophilic PVDF membranes for effective separation of water-in-oil emulsions with high flux. Adv. Mater..

[22] Wandera D., Wickramasinghe S.R., Husson S.M. (2011). Modification and characterization of ultrafiltration membranes for treatment of produced water. J. Membr. Sci..

[23] Kim D., Livazovic S., Falca G., Nunes S.P. (2018). Oil–water separation using membranes manufactured from cellulose/ionic liquid solutions. ACS Sustain. Chem. Eng..

[24] Usman J., Othman M.H.D., Ismail A.F., Rahman M.A., Jaafar J., Raji Y.O., Said K.A.M. (2021). An overview of superhydrophobic ceramic membrane surface modification for oil-water separation. J. Mater. Res. Technol..

[25] Zioui D., Salazar H., Aoudjit L., Martins P.M., Lanceros-Méndez S. (2019). Polymer-based membranes for oily wastewater remediation. Polymers.

[26] Zioui D., Martins P.M., Aoudjit L., Salazar H., Lanceros-Méndez S. (2023). Wastewater Treatment of Real Effluents by Microfiltration Using Poly (vinylidene fluoride–hexafluoropropylene) Membranes. Polymers.

[27] Peer P., Polaskova M., Musilova L. (2019). Superhydrophobic poly (vinyl butyral) nanofibrous membrane containing various silica nanoparticles. J. Text. Inst..

[28] Zhang W., Sun G., Wang Y., Han W., Zhang Y., Hu W., Xiao C. (2022). Superhydrophobic and Breathable Polyacrylonitrile/Silica/Perfluoroalkyl Ethyl Methacrylate Nanofiber Membranes Prepared by Solution Blow Spinning. ACS Omega.

[29] Jiang S., Meng X., Chen B., Wang N., Chen G. (2020). Electrospinning superhydrophobic–superoleophilic PVDF-SiO_2_ nanofibers membrane for oil–water separation. J. Appl. Polym. Sci..

[30] Ju J., Wang T., Wang Q. (2015). A facile approach in fabricating superhydrophobic and superoleophilic poly (vinylidene fluoride) membranes for efficient water–oil separation. J. Appl. Polym. Sci..

[31] Kango S., Kalia S., Celli A., Njuguna J., Habibi Y., Kumar R. (2013). Surface modification of inorganic nanoparticles for development of organic–inorganic nanocomposites—A review. Prog. Polym. Sci..

[32] Taurozzi J.S., Arul H., Bosak V.Z., Burban A.F., Voice T.C., Bruening M.L., Tarabara V.V. (2008). Effect of filler incorporation route on the properties of polysulfone–silver nanocomposite membranes of different porosities. J. Membr. Sci..

[33] Muhamad M.S., Salim M.R., Lau W.J. (2015). Surface modification of SiO_2_ nanoparticles and its impact on the properties of PES-based hollow fiber membrane. RSC Adv..

[34] Qing W., Shi X., Deng Y., Zhang W., Wang J., Tang C.Y. (2017). Robust superhydrophobic-superoleophilic polytetrafluoroethylene nanofibrous membrane for oil/water separation. J. Membr. Sci..

[35] Wang S., Li M., Lu Q. (2010). Filter paper with selective absorption and separation of liquids that differ in surface tension. ACS Appl. Mater. Interfaces.

[36] Zhou Z., Wu X.F. (2015). Electrospinning superhydrophobic–superoleophilic fibrous PVDF membranes for high-efficiency water–oil separation. Mater. Lett..

[37] Ahmed F.E., Lalia B.S., Hashaikeh R. (2015). A review on electrospinning for membrane fabrication: Challenges and applications. Desalination.

[38] Kravets L., Gainutdinov R., Gilman A., Yablokov M., Satulu V., Mitu B., Dinescu G. (2018). Morphology and Contact Properties of Polytetrafluoroethylene-Like Films Deposited onto Track-Etched Membrane Surface in Vacuum. Plasma Phys. Technol..

[39] Pierson H.O. (1999). Handbook of Chemical Vapor Deposition: Principles, Technologies and Applications.

[40] Kravets L.I., Yarmolenko M.A., Gainutdinov R.V., Satulu V., Mitu B., Dinescu G. (2021). Formation of hydrophobic polymer coatings on the track-etched membrane surface. Int. J. Phys. Conf. Ser..

[41] Kravets L.I., Yarmolenko M.A., Rogachev A.A., Gainutdinov R.V., Gilman A.B., Altynov V.A., Lizunov N.E. (2020). Formation of Superhydrophobic Coatings on the Track-Etched Membrane Surface by the Method of Electron-Beam Deposition of Polymers in Vacuum. Inorg. Mater. Appl. Res..

[42] Martin I., Bertin M., Domaracka A., Azria R., Illenberger E., Lafosse A. (2008). Chemistry induced by low-energy electrons in condensed multilayers of pure small organic acids. Int. J. Mass Spectrom..

[43] Sala L., Szymańska I.B., Dablemont C., Lafosse A., Amiaud L. (2018). Response under low-energy electron irradiation of a thin film of a potential copper precursor for focused electron beam induced deposition (FEBID). Beilstein J. Nanotechnol..

[44] Thorman R.M., TP R.K., Fairbrother D.H., Ingólfsson O. (2015). The role of low-energy electrons in focused electron beam induced deposition: Four case studies of representative precursors. Beilstein J. Nanotechnol..

[45] Rogachev A.A., Yarmolenko M.A., Rogachev A.V., Xiaohong J., Cao H., Lysenko E.N., Surzhikov A.P. (2019). Structure and electrical properties of polyaniline-based copper chloride or copper bromide coatings deposited via low-energy electron beam. Appl. Surf. Sci..

[46] Liu Y., Qin X., Rogachev A.V., Rogachev A.A., Kontsevaya I.I., Pyzh A.E., Yarmolenko M.A. (2021). Structure and properties of microcellulose-based coatings deposited via a low-energy electron beam and their effect on the properties of onto wound dressings. Carbohydr. Polym. Technol. Appl..

[47] Hliavitskaya T., Plisko T., Bildyukevich A., Lipnizki F., Rodrigues G., Sjölin M. (2020). Modification of PES ultrafiltration membranes by cationic polyelectrolyte Praestol 859: Characterization, performance and application for purification of hemicellulose. Chem. Eng. Res. Des..

[48] Huotari H.M., Huisman I.H., Trägårdh G. (1999). Electrically enhanced crossflow membrane filtration of oily waste water using the membrane as a cathode. J. Membr. Sci..

[49] Dmitrenko M., Kuzminova A., Zolotarev A., Markelov D., Komolkin A., Loginova E., Penkova A. (2022). Modification strategies of polyacrylonitrile ultrafiltration membrane using TiO_2_ for enhanced antifouling performance in water treatment. Sep. Purif. Technol..

[50] Mahdavi H., Karami M., Heidari A.A. (2021). Preparation of mixed matrix membranes made up of polysulfone and MIL-53 (Al) nanoparticles as promising membranes for separation of aqueous dye solutions. Sep. Purif. Technol..

[51] Zhang B., Shi W., Yu S., Zhu Y., Zhang R., Tay J.H. (2019). Adsorption of anion polyacrylamide from aqueous solution by polytetrafluoroethylene (PTFE) membrane as an adsorbent: Kinetic and isotherm studies. J. Colloid Interface Sci..

[52] Ahmad A.L., Abdulkarim A.A., Ooi B.S., Ismail S. (2013). Recent development in additives modifications of polyethersulfone membrane for flux enhancement. Chem. Eng. J..

[53] Bildyukevich A.V., Hliavitskaya T.A., Kavalenka M.N. (2020). The Modification of polyethersulfone membranes using a Synperonic F108 block copolymer and their application for the fractionation of thermomechanical pulp mill process water. Membr. Membr. Technol..

[54] Plisko T.V., Bildyukevich A.V., Burts K.S., Ermakov S.S., Penkova A.V., Kuzminova A.I., Ulbricht M. (2020). One-step preparation of antifouling polysulfone ultrafiltration membranes via modification by a cationic polyelectrolyte based on polyacrylamide. Polymers.

[55] Plisko T.V., Bildyukevich A.V., Burts K.S., Hliavitskaya T.A., Penkova A.V., Ermakov S.S., Ulbricht M. (2020). Modification of polysulfone ultrafiltration membranes via addition of anionic polyelectrolyte based on acrylamide and sodium acrylate to the coagulation bath to improve antifouling performance in water treatment. Membranes.

